# Blockade of Immunosuppressive Cytokines Restores NK Cell Antiviral Function in Chronic Hepatitis B Virus Infection

**DOI:** 10.1371/journal.ppat.1001227

**Published:** 2010-12-16

**Authors:** Dimitra Peppa, Lorenzo Micco, Alia Javaid, Patrick T. F. Kennedy, Anna Schurich, Claire Dunn, Celeste Pallant, Gidon Ellis, Pooja Khanna, Geoffrey Dusheiko, Richard J. Gilson, Mala K. Maini

**Affiliations:** 1 Division of Infection and Immunity, UCL, London, United Kingdom; 2 Centre for Sexual Health and HIV Research, UCL, London, United Kingdom; 3 Centre for Digestive Disease, Barts and The London School of Medicine and Dentistry, London, United Kingdom; 4 Centre for Hepatology, Hampstead Campus, Royal Free & University College Medical School, London, United Kingdom; The Scripps Research Institute, United States of America

## Abstract

NK cells are enriched in the liver, constituting around a third of intrahepatic lymphocytes. We have previously demonstrated that they upregulate the death ligand TRAIL in patients with chronic hepatitis B virus infection (CHB), allowing them to kill hepatocytes bearing TRAIL receptors. In this study we investigated whether, in addition to their pathogenic role, NK cells have antiviral potential in CHB. We characterised NK cell subsets and effector function in 64 patients with CHB compared to 31 healthy controls. We found that, in contrast to their upregulated TRAIL expression and maintenance of cytolytic function, NK cells had a markedly impaired capacity to produce IFN-γ in CHB. This functional dichotomy of NK cells could be recapitulated *in vitro* by exposure to the immunosuppressive cytokine IL-10, which was induced in patients with active CHB. IL-10 selectively suppressed NK cell IFN-γ production without altering cytotoxicity or death ligand expression. Potent antiviral therapy reduced TRAIL-expressing CD56^bright^ NK cells, consistent with the reduction in liver inflammation it induced; however, it was not able to normalise IL-10 levels or the capacity of NK cells to produce the antiviral cytokine IFN-γ. Blockade of IL-10 +/− TGF-β restored the capacity of NK cells from both the periphery and liver of patients with CHB to produce IFN-γ, thereby enhancing their non-cytolytic antiviral capacity. In conclusion, NK cells may be driven to a state of partial functional tolerance by the immunosuppressive cytokine environment in CHB. Their defective capacity to produce the antiviral cytokine IFN-γ persists in patients on antiviral therapy but can be corrected in vitro by IL-10+/− TGF-β blockade.

## Author Summary

Hepatitis B virus (HBV) infection is responsible for more than a million deaths annually as a result of the immune-mediated chronic liver damage it induces. One of the key immune players in the liver is the natural killer (NK) cell, which we have recently found can cause liver damage in HBV infection. Here we address the antiviral potential of NK cells in the HBV-infected liver and demonstrate that they have a specific impairment in their ability to produce the cytokine IFN-γ, which could limit their capacity to control HBV. We find that the potent antiviral drugs currently being used to treat HBV infection are unable to fully reverse this NK cell functional defect. We define a role for the immunosuppressive cytokine environment in HBV in down-regulating NK cell antiviral function, which can be restored by specific blockade of IL-10 and TGF-β. This work therefore highlights a mechanism contributing to the failure of immune control in chronic HBV infection, paving the way to new therapeutic options.

## Introduction

NK cells constitute a major cellular arm of the innate immune system and, as such, have been viewed as most relevant in the setting of the initial response to an acute infection. However, they may also be appropriately or inappropriately activated to exert effector function when persistent infection and its pathological sequelae become established. Their role may be particularly important in patients with CHB, in whom the virus-specific CD8 T cell arm of protection is markedly diminished and dysfunctional [1], [2].

NK cells are greatly enriched in the liver, the site of HBV replication[3], [4]. We have previously demonstrated an increase in activated CD56^bright^ NK cells in the livers of patients undergoing flares of eAg-negative CHB. This subset can be induced to express TNF-related apoptosis-inducing ligand (TRAIL), which is able to kill hepatocytes that have upregulated death-inducing TRAIL receptors, thereby contributing to liver inflammation in CHB[4]. The CD56^bright^ subset can also be a potent source of cytokines such as IFN-γ[5], [6], a key cytokine shaping adaptive immunity and the delicate balance between protective and pathogenic responses. IFN-γ can clear HBV-infected hepatocytes through non-cytolytic mechanisms[7], [8]. NK cell-derived IFN-γ could therefore constitute a vital antiviral mechanism in the liver, where hepatocytes are relatively resistant to the cytolytic mechanisms of perforin and granzyme production[9].

The intensity and quality of NK cell effector function is determined by the balance of activatory and inhibitory signals through their array of receptors (NK-R), in addition to the influences exerted by the cytokine microenvironment. The TRAIL pathway of NK cell-mediated hepatocyte killing can be driven by the cytokines IFN-α and IL-8, induced during flares of CHB[4]. Similarly, NK cells in HCV infection can be polarised towards cytolysis and expression of TRAIL as a result of exposure to endogenous[10] or therapeutic[11] IFN-α. Conversely, intrahepatic NK cell function can be down-regulated by the immunosuppressive cytokine IL-10 produced by Kupffer cells[12]. In addition, a role for IL-17 in curtailing NK cell function was recently demonstrated in disseminated vaccinia virus infection of mice with pre-existing dermatitis[13]. In this study we have investigated cytokine-driven modulation of IFN-γ production by NK cells in patients with CHB and explored the potential to restore their non-cytolytic antiviral function.

## Results

### Expansion of the CD56^bright^ subset of NK cells in CHB

**Figure 1:**
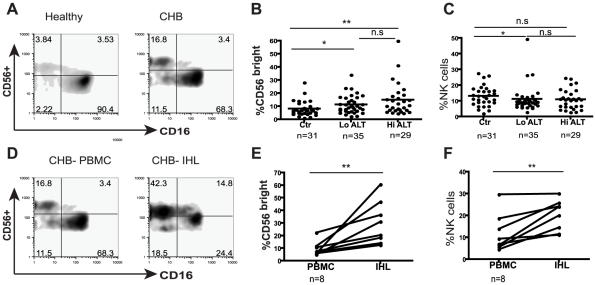
NK cell frequency and altered subset distribution in the periphery and intrahepatic compartment. (**A**) Representative density plots gated on CD3- PBMC and co-stained for CD56 and CD16 to identify NK cells from a healthy control and a CHB patient. (**B**) Summary data of the proportions of CD56^bright^ subset in the periphery of CHB patients with low ALT (n = 35, ALT <50IU/L, median 34) compared to high ALT (n = 29, median ALT 112) and healthy controls (n = 31). (**C**) Frequency of circulating NK cells in CHB patients with low ALT and high ALT and healthy controls. (**D**) Density plots of NK cells from peripheral blood and intrahepatic lymphocytes from a representative CHB patient. (**E**) Paired cumulative results of peripheral and intrahepatic CD56^bright^ NK cells frequencies from 8 patients with CHB. (**F**) NK cell frequency in peripheral blood and intrahepatic compartment from 8 patients with CHB with paired samples. The non-parametric Mann-Whitney U test was used to compare data between groups and the Wilcoxon signed rank test was used between paired variables. *p<0.05 or ** p<0.01 designates values that differ significantly between groups. Ctr  =  healthy controls.

To explore NK cell effector potential in the setting of persistent HBV infection, we first analysed the frequency of CD56^bright^(CD16^dim^/^neg^) and CD56^dim^(CD16^pos^) NK cell subsets in 64 patients with CHB compared to 31 healthy age-matched controls (Table 1). The proportion of circulating CD56^bright^ NK cells was significantly increased in patients with CHB (representative FACS plots Fig1a, summary data Fig1b), with a tendency to further increases in those with liver inflammation (Fig1b). There was a trend for the percent of circulating NK cells to decrease in CHB (Fig 1c) but the absolute number of circulating CD56^bright^ NK cells was still significantly increased (*p<0.05* data not shown).

**Table 1:** Characteristics of study population.

	Healthy Controlsn = 31	HBV PatientsHigh ALTn = 29	HBV PatientsLow ALTn = 35	Treatment Group (Lamivudine and Adefovir)n = 22
Age, years: median (range)	30 (18–52)	43.5 (23–65)	32 (23–65)	43 (18–70)
Sex (Female:male)	14∶17	14∶15	16∶19	5∶17
ALT IU/L: median (range)	na	112 (57–604)	34 (10–47)	25 (18–70)
HBV DNA IU/mL: median (range)	na	1,546,000 (1150–2.9×10^8^)	870 (100–3.3×10^8^)	<100
HBeAg+	na	18	3	6

na = not applicable.

To determine whether there was a further enrichment of this immunoregulatory CD56^bright^ NK cell subset at the site of viral replication, we compared the proportions in intrahepatic and circulating lymphocytes. In all eight patients with CHB from whom paired samples were available, the percent of CD56^bright^ of total NK cells was higher in the intrahepatic compared to peripheral compartment (Fig1d,e). Since NK cells make up a significantly greater proportion of intrahepatic than circulating lymphocytes in these patients (Fig 1f), this corresponds to a substantial enrichment of CD56^bright^ NK cells in the liver.

### Impaired non-cytolytic antiviral potential of NK cells in CHB

**Figure 2:**
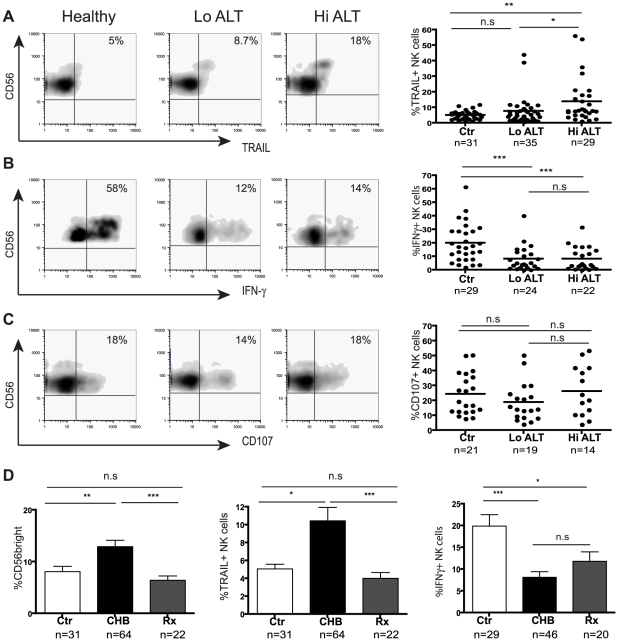
Skewed NK cell effector function in CHB is only partially corrected during therapy. (Panels **A–C**) Representative density plots from a healthy control and HBV patients with low ALT (ALT <50 IU/L, median 33) and raised ALT (ALT>50 IU/L, median 112) and summary data for TRAIL expression, IFN-γ production and CD107 expression. (**D**) Summary bar charts of CD56^bright^ proportions, NK cell TRAIL expression and NK cell IFN-γ production from healthy, CHB and patients on antiviral therapy. Results are expressed as mean ± SEM. Rx  =  treated patients. *p<0.05, **p<0.01, ***p<0.001 by Mann-Whitney test.

We have previously shown that the CD56^bright^ subset of NK cells can mediate hepatocyte apoptosis through expression of the death ligand TRAIL in flares of eAg-negative CHB[4]. In this cohort of patients we confirmed an increase in TRAIL expression (largely on the CD56^bright^ subset, Fig 2a representative plots) in patients with either eAg+ or eAg- CHB who had evidence of liver inflammation (Fig2a summary data).

The CD56^bright^ subset of NK cells can also be a potent source of IFN-γ[14], a cytokine that has direct non-cytolytic antiviral effects on HBV replication [7], [8] and can promote adaptive immune responses[6]. Despite the enrichment of CD56^bright^ NK cells in CHB, we found that they had an impaired capacity to produce IFN-γ (representative plots, Fig2b). There was a significant reduction in production of IFN-γ by NK cells from 46 patients with CHB compared to 29 healthy controls (Fig2b). This reduction was seen irrespective of disease activity (liver inflammation Fig2b, viral load or eAg status, data not shown) or method of NK cell stimulation (IL-12/IL-18 (Fig2b), IL-12/IL-15, K562 with IL-12/IL-18 or PMA/ionomycin, data not shown). Both the CD56^bright^ subset and the CD56^dim^ subset (that has recently been recognised to also make a contribution to cytokine production[15]) showed significantly impaired IFN-γ production (FigS1a). Similarly, CD56^bright^ and CD56^dim^ NK cells in CHB showed a trend to produce less TNF-α, despite the strong stimulus required to reliably elicit this cytokine (FigS1b). Simultaneous assessment of IFN-γ and TNF-a production showed a significant reduction in dual producing NK cells in CHB (FigS1c).

To assess NK cell cytolytic potential, we determined their capacity to degranulate as evidenced by CD107 expression following stimulation with K562 target cells and cytokines. There was no significant difference in NK cell degranulation potential in 33 patients with CHB compared to 21 controls (Fig2c). Differential analysis by NK cell subset or by patient disease status did not show any differences (data not shown). NK cells in CHB were therefore biased towards cytolytic and death-ligand mediated effector functions and defective IFN-γ production.

To determine the potential of potent antiviral treatment to correct this bias in NK cell effector function, we studied a group of 22 patients with HBV viraemia well-suppressed on a combination of Lamivudine and Adefovir. Upon viral suppression and normalisation of liver inflammatory markers, there was no significant change in the percent of NK cells (FigS2a), but the proportion of CD56^bright^ NK cells decreased to levels observed in healthy controls (Fig2d); in line with this, NK cell TRAIL expression reduced to baseline levels (Fig2d). However NK cell IFN-γ production was only partially augmented upon antiviral treatment (mainly CD56^dim^ subset, FigS2b) and remained significantly lower than that in healthy controls (Fig2d).

### IL-10 is induced in CHB and recapitulates the NK cell defect in IFN-γ production

**Figure 3:**
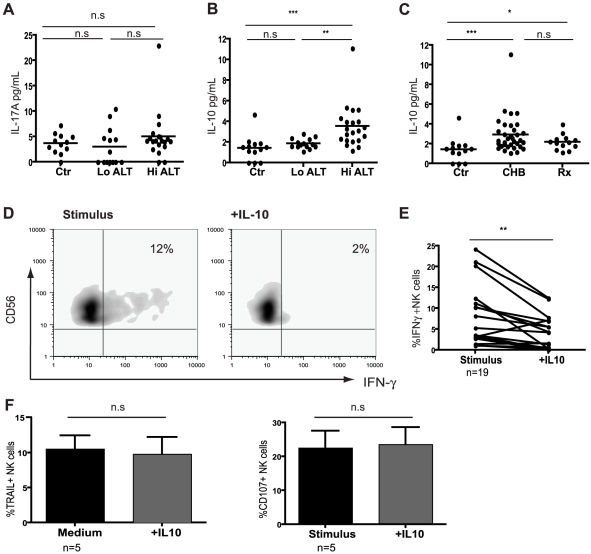
IL-10 is elevated in CHB and suppresses NK cell IFN-γ production. (**A**) Levels of cytokines IL-17A and (**B**) IL-10 determined using Cytometric Bead Arrays flex sets using sera from 13 healthy controls, 14 low ALT (median ALT 35, all eAg-) and 21 high ALT patients (median ALT 115, 13eAg-). (**C**) Cumulative IL-10 results including therapy group (n = 13, median ALT 25)****. (**D**) Representative density plots of the effect of exogenous IL-10 on IFN-γ production by NK cells from a CHB patient and (**E**) paired cumulative results from 19 CHB patients. (**F**) Summary bar charts of the effect of exogenous IL-10 on the expression of TRAIL and CD107 in 5 CHB patients. Results are paired and expressed as mean ± SEM. Stimulus  =  IL12+IL18. Significance determined by the Mann-Whitney test for comparison between groups and the Wilcoxon signed rank test for paired data, *p<0.05, **p<0.01, ***p<0.001.

Effector function of NK cells is tightly regulated by the cytokine milieu and their production of IFN-γ can be inhibited by immunosuppressive cytokines such as IL-10[12], [16] and IL-17[13]. The levels of IL-17A were not elevated in sera from patients with CHB compared to controls (Fig3a). In contrast, circulating concentrations of IL-10 were significantly increased in patients with active HBV disease (Fig3b,c by CBA, confirmed by ELISA, data not shown), correlating with viral load (r = 0.48, p = 0.002) and ALT (r = 0.37, p = 0.03). IL-10 levels showed a trend to decrease on antiviral treatment but remained significantly higher than in controls (Fig3c), consistent with the limited restoration of NK cell IFN-γ production in these patients.

To test whether IL-10 could induce the defect in NK cell IFN-γ production seen in CHB, we re-assessed NK cell effector function with or without the addition of exogenous IL-10. IL-10 significantly suppressed NK-cell derived IFN-γ (Fig3d), particularly in those patients in whom it was not already substantially reduced (Fig3e, and in healthy controls, data not shown). By contrast, IL-10 had no effect on cytolytic ability or TRAIL phenotype (Fig3f) and did not affect the percent of NK cells (FigS3a). The ability of IFN-α to further induce NK cell TRAIL expression *in vitro* [4] was also not abrogated by IL-10 (data not shown). The effect of IL-10 was consistent but more modest on purified NK cells (FigS3b), suggesting that some of its suppressive activity on NK cells is mediated indirectly via other constituents such as APCs. The contrasting effects of IL-10 on TRAIL and IFN-γ expression represented differential regulation of these effector functions in the same NK cells rather than the emergence of two distinct subsets. The small population of TRAIL-expressing NK cells present in healthy donors were at least as able to produce IFN-γ as the rest of the NK cell population (FigS3c). The addition of exogenous IL-10 suppressed IFN-γ in NK cells regardless of their TRAIL expression (FigS3c). In line with this, gating on the expanded population of TRAIL-expressing NK cells found in CHB demonstrated that their IFN-γ-producing capacity was no more reduced than that of the non-TRAIL-expressing fraction (FigS3d).

### Restoration of NK cell IFN-γ production upon blockade of immunosuppressive cytokines

**Figure 4:**
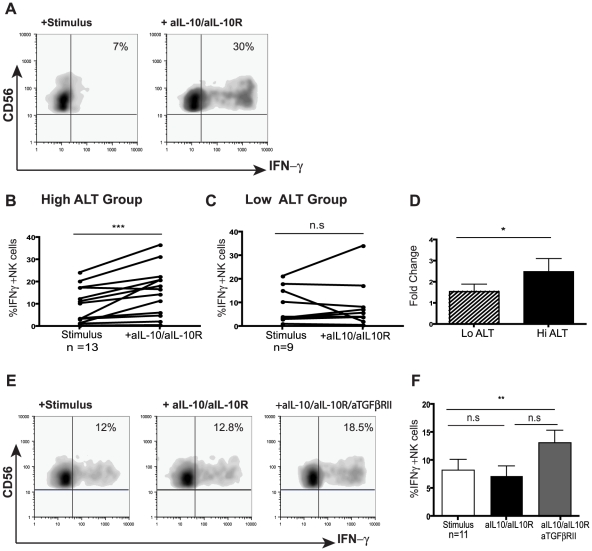
IL-10 blockade alone or in combination with TGFβRII blocking restores NK cell IFN-γ production. (**A**) Representative density plot from a CHB patient of peripheral NK cell IFN-γ production in the presence of anti-IL-10 and anti-IL10 receptor blocking mAb****. (**B**) Paired summary data from CHB patients with either active disease (High ALT median 104, n = 13) or (**C**) inactive disease (Low ALT median 33, n = 9). (**D**) Fold change in IFN-γ produced by total NK cells following IL-10 blockade in both groups of patients. (**E, F**) Representative density dot plots from a CHB patient and summary bar chart of paired results from 11 patients (n = 11 median ALT 42) of NK cell IFN-γ production following IL-10 blockade alone or in combination with anti-TGFβRII blocking antibodies. Stimulus  =  IL12+IL18. Significance determined by the Mann-Whitney test for comparison between groups and the Wilcoxon signed rank test for paired data, *p<0.05, **p<0.01.

Since IL-10 was induced in CHB and exogenous IL-10 was able to mimic the selective suppression of NK cell effector function, we next investigated the potential to restore NK cell IFN-γ production by IL-10 blockade. Addition of antiIL10/IL10-R blocking mAbs restored the ability of both CD56^bright^ and CD56^dim^ NK cells from patients with active CHB to produce IFN-γ (mean 2.5 fold increase, Fig4a,b,d). The majority of patients without biochemical evidence of liver inflammation (and with low viral loads) did not respond to this strategy (Fig4c,d), in line with their lower levels of circulating IL-10 (Fig3b). A subset of those patients failing to respond to IL-10 blockade did show recovery of NK cell IFN-γ production following blockade of both IL-10 and TGFβ, another immunosuppressive cytokine known to be able to inhibit NK cell production (Fig4e,f).

**Figure 5:**
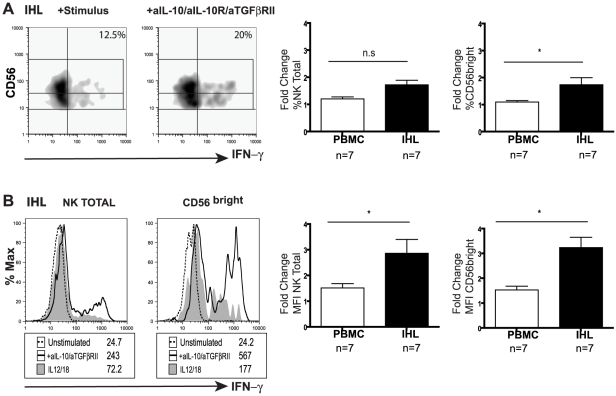
Blockade of IL10/TGF enhances intrahepatic NK cell IFN-γ production. (**A**) Representative density plots and (**B**) histograms for total intrahepatic NK cell and CD56^bright^ subset IFN-γ production upon blockade with anti-lL-10, anti-IL10 receptor and anti-TGFβRII blocking antibodies. Paired summary bar charts of fold change increase in the percentage and mean fluorescence intensity (MFI) of NK total and CD56^bright^ IFN-γ+ cells in the periphery and intrahepatic compartment of 7 CHB (median ALT 56). Results are expressed as mean ± SEM. Stimulus  =  IL12+IL18. *p<0.05 by Wilcoxon signed rank test.

To investigate whether the suppression of NK cell IFN-γ was maintained at the site of HBV replication, paired liver and blood samples from eight patients with CHB were examined (Table 2). CD56^bright^ NK cell IFN-γ production showed a trend to be even lower in the liver than the periphery of patients with CHB (FigS4a). Levels of intrahepatic NK cell IFN-γ production did not significantly correlate with levels of ALT (FigS4b), viral load or liver histology in this small sample of patients, only one of whom had histological evidence of significant liver inflammation (Table 2). Due to limited cell numbers, individual cytokine blockade could not be performed but dual IL-10/TGFβRII blockade reconstituted the proportion of NK cells able to produce IFN-γ (%positive, Fig5a) and increased their level of IFN-γ production (MFI, Fig5b). The fold increase in the capacity of CD56^bright^ NK cells to secrete IFN-γ upon IL-10/TGFβ blockade was greater in the liver than the periphery (Fig5a,b).

**Table 2:** Patient characteristics with available liver biopsy specimens.

Patients n = 8	AgeMedian 35.5Range 24–66	SexM:F6∶2	HBeAg+ 2/6	HBV DNA (IU/mL) Median 66,879Range 646–1.2×10^6^	ALT (IU/L) Median 56 Range 15–113	Necro-inflammatory score	Modified ISHAK Stage Fibrosis
**Pt1**	25	F	Pos	113,757	113	2/18	1/6
**Pt2**	32	M	Pos	310,000	63	4/18	3/6
**Pt3**	49	M	Neg	700,000	15	na	1/6
**Pt4**	40	F	Neg	20,000	26	3/18	1/6
**Pt5**	24	M	Neg	947	86	2/18	1/6
**Pt6**	66	M	Neg	646	56	3/18	1/6
**Pt7**	39	M	Neg	6500	26	3/18	1/6
**Pt8**	27	M	Neg	1.2×10^6^	56	3/18	1/6

na = not available.

## Discussion

Accumulating evidence points to a contribution of NK cells in the battle to control persistent intracellular pathogens[6], [17], [18]. Although NK cells have been considered part of the innate immune response, recent data have suggested that they can possess properties previously ascribed to the adaptive arm, including the capacity to develop memory and tolerance[19], [20], [21]. In this study we show that NK cells can develop selective defects in antiviral function in the setting of chronic infection and inflammation, reminiscent of the hierarchical loss of effector function manifested by exhausted T cells[22].

Just as T cell defects have been attributed to excessive antigenic stimulation, functional impairment of NK cells has been ascribed to excessive stimulatory signals through the activating receptor NKG2D, resulting in its down-modulation[19], [20]. This is a plausible mechanism in CHB since data from transgenic mice suggest that HBV can upregulate the intrahepatic expression of NKG2D ligands[23]. However, a recent study and our unpublished data do not support this mechanism, showing no down-regulation of NKG2D or consistent changes in other NK cell receptors that could account for the NK cell impairment seen in CHB[24]. Instead, our data suggest that the selective NK cell functional defects seen in this infection may be attributable to the immunosuppressive cytokine milieu.

Our analysis of NK cell effector potential in a large cohort of patients with CHB revealed preservation of cytolytic capacity and an increase in TRAIL-bearing CD56^bright^ NK cells. Despite this increase in the subset of NK cells that are usually the most potent source of cytokines[14], there was a decrease in the overall NK cell capacity to produce IFN-γ. Such divergence of effector function is in line with the recent finding that cytokines are trafficked and secreted via completely different pathways to cytotoxic granules in NK cells[25]. Consistent with these distinct trafficking pathways, separate signalling pathways have been shown to control the release of cytokines and cytotoxic granules in NK cells[26], [27]. Unique molecular switches are starting to be identified that couple NK cell receptor signalling with the generation of cytokines rather than cytotoxic functions[28], [29]. It is therefore conceivable that a pathway specific to NK cell cytokine production is dysregulated in patients with CHB.

The immunosuppressive cytokine IL-10 has been shown to specifically impair NK cell IFN-γ production[30], in contrast with IL-17 and excessive NKG2D signalling, both of which result in down-modulation of all NK cell effector functions[13], [20]. The liver is an immunotolerant organ, predisposed to the production of immunosuppressive cytokines; down-regulation of intrahepatic NK cell IFN-γ production has been linked to the local release of IL-10 by Kupffer cells[12], [31]. We found that exposure of NK cells to IL-10 *in vitro* was able to recapitulate the selective reduction in IFN-γ production noted in patients with CHB. Furthermore, its blockade was able to restore the capacity of NK cells from patients with active HBV infection to produce IFN-γ. IL-10 was not able to inhibit cytotoxic degranulation and could not overcome the capacity of IFN-α to induce TRAIL, in line with the maintenance of these pathogenic functions of NK cells in CHB. IL-10 was consistently modestly elevated in the serum of patients with CHB, but would be expected to be at higher concentrations at the site of infection in the liver and in close proximity to the cells from which it is released. NK cells themselves can produce IL-10[14], [32] to allow auto-suppression, but in the HBV-infected liver there are a number of other candidate cellular sources and there is likely to be a complex regulatory network involved in maintaining its production, as recently described in HIV infection[33].

We recently reported a transient induction of IL-10 in early acute HBV infection that was temporally associated with a transient suppression of the capacity of NK cells to produce IFN-γ, coincident with the increase in viraemia and production of viral antigens[16]. In our cohort of patients with CHB it was difficult to distinguish the influence of viraemia or liver inflammation, since both were increased in patients with elevated levels of IL-10. Future study of a group of patients with high viral load but normal ALT (immunotolerant phase) could help to dissect the role of these factors. The fact that NK cell IFN-γ production and IL-10 levels were not significantly normalised by potent antiviral therapy suggests that the continued secretion of high levels of HBV proteins in these patients may play a role. In patients with low level CHB without evidence of liver inflammation, IL-10 was not elevated and its blockade alone could not rescue NK function, which instead required additional TGF-β blockade. TGF-β is another immunosuppressive cytokine that characterises the tolerising liver environment and has been shown to be increased in CHB[34]. TGF-β has been shown to be an alternative key regulator of the capacity of human NK cells to produce IFN-γ, suppressing IFN-γ and T-bet via Smad2/3/4[35].

The collective action of TGF-β and IL-10 may represent an important feedback mechanism to limit exuberant immune responses and tissue immunopathology in a vital organ like the liver. However, in the context of chronic infections, elevated levels may attenuate immune responses sufficiently to contribute to the failure of resolution of infection. A role for IL-10 in persistent viral infection has been highlighted recently by studies showing that blockade of the IL-10 receptor is associated with resolution of LCMV infection[36], [37]. Genetic studies have also highlighted the importance of IL-10 in the antiviral response to HBV; polymorphisms of the IL-10 promoter resulting in elevated IL-10 production are associated with viral persistence, increased disease severity and progression[38], [39].

Our data suggest that immunosupressive cytokines may polarise NK cells in CHB, having no effect on their expression of death ligands and cytolytic granules but inhibiting IFN-γ production. NK cells expressing death ligands like TRAIL would only be able to have a direct antiviral effect at the expense of liver damage. The decline in liver inflammation seen on antiviral treatment is compatible with the reduction in TRAIL-expressing CD56^bright^ NK cells that we noted in this setting. However, potent antiviral therapy was unable to significantly restore the capacity of NK cells to produce IFN-γ, which would therefore retain an impaired capacity for non-cytolytic clearance of HBV from hepatocytes and boosting of adaptive immune responses. Our findings raise the possibility of immunotherapeutic targeting of IL-10 and TGF-β in CHB, with the caveat that these cytokines govern a critical balance between impeding pathogen clearance and restraining immunopathology.

## Materials and Methods

### Ethics statement

Clinical assessment and blood sampling were performed during routine hepatitis clinics, with written informed consent and local ethical board approval of the Royal Free Hospital, the Royal London Hospital and Camden Primary Care Ethics Review Board.

### Patients and healthy subjects

All patients were anti-Hepatitis C- and anti-Human Immunodeficiency Virus-antibody negative and treatment naïve with the exception of a sub-group of 22 patients suppressed on a combination of Lamivudine and Adefovir. Patient characteristics are included in Table 1. Paired peripheral blood and liver biopsy specimens (surplus to diagnostic requirements) were obtained from 8 CHB-infected patients (Table 2).

### Isolation and storage of PBMC and Intrahepatic lymphocyte isolation

Peripheral blood mononuclear cells (PBMC) were isolated by gradient centrifugation on Ficoll-Hypaque and frozen or immediately studied as described later. Sera were collected and frozen for later use. Intrahepatic lymphocytes were isolated as previously described[4].

### Extracellular staining and flow cytometric analysis

For phenotypic analysis, PBMC isolated from HBV patients and healthy donors were stained with fluorochrome-conjugated antibodies to CD3-Cy5.5/PerCP, CD56-FITC, CD16-APC, and TRAIL-PE or isotype matched controls (BD Biosciences, Cowley, U.K.). In selected experiments TRAIL expression was determined following overnight incubation with 50 ng/mL of rhIL-10 (eBioscience). PBMC were acquired on a FACS Calibur flow cytometer (Becton Dickinson) and analysed using Flowjo analysis software (Treestar).

### Cytokine production by intracellular staining

As previously described[16], PBMC were incubated with 50 ng/mL of rhIL-12 (Miltenyi) and rhIL-18 (R&D Systems, Abingdon, U.K.) for 21 hours at 37°C. 1mM monensin (Sigma-Aldrich, Gillingham, U.K.) was added for the final 3 hours. Cells were fixed and permeabilised followed by intracellular staining for IFN-γ-PE (R&D systems). Where indicated the same experiments were performed in the presence of rhIL-10 (50ng/mL), or blocking antibodies to anti-IL10 (5 µg/mL) (eBioscience) and anti-IL-10R (10 µg/mL) alone or in combination with antiTGFβRII (10 µg/mL) (BD Biosciences). NK IFN-γ production was determined by subtracting baseline IFN-γ production from that observed after cytokine or antibody treatment. NK cells from PBMC of a randomly selected group of patients were isolated (>96% purity and viability) (Miltenyi Biotec, Germany, NK isolation kit) to assess the effect of exogenous IL-10 on IFN-γ production.

For TNF-α production, PBMC were stimulated with phorbol myristate acetate (PMA) (3 ng/mL) and ionomycin (100 ng/mL) (Sigma) for 3 hours; 1mM monensin (Sigma-Aldrich, Gillingham, U.K.) was added for the final 2 hours. Cells were then stained with the same antibody combination used for phenotyping prior to permealisation and intracellular staining for TNF-α. In selected experiments NK cell TNF-α and IFN-γ co-expression was assessed following PMA/I stimulation.

### CD107 degranulation assay

As previously described[16] **,** PBMC were incubated with K562 cells (5∶1 E:T ratio) for 3 hours at 37°C following overnight stimulation with a combination of rhIL-12/rhIL-18 or medium alone in the presence or absence of rh-IL10. CD107a-PE antibody (BD Biosciences, Cowley, U.K.) was added at the time of stimulation with target cells and 1mM monensin was added during the last two hours of the incubation prior to staining and acquisition.

### Determination of serum cytokine concentrations by Cytometric Bead Array (CBA)

CBA flex-sets were used for the determination of IL-10, IL-17 (BD Biosciences, Cowley, U.K) according to manufacturers’ protocols for serum samples.

### Statistical analysis

Statistical significance was performed between paired samples using the Wilcoxon signed rank test and between HBV patients and healthy controls using the Mann-Whitney U test. Correlations between variables were evaluated with the Spearman rank correlation test. P<0.05 was considered to be significant for all tests.

## Supporting Information

Figure S1 Summary bar charts comparing production of (**A**) IFN-γ and (**B**) TNF-α from NK total and NK cell subsets in healthy controls and CHB patients. (C) 10 healthy controls and 12 CHB patients were evaluated for the co-production of TNF-α and IFN-γ following stimulation with PMA/I. Summary bar charts show the percentage of total NK cells that are single positive for IFN-γ, TNF-α and double positive for IFN-γ/TNF-α. *P<.05, **P<.01, ***P<.001 by Mann-Whitney test.
(0.48 MB EPS)

Figure S2 (**A**) Frequencies of circulating NK cells and (**B**) summary bar chart comparing production of IFN-γ from NK total and NK cell subsets in healthy controls (n = 29), CHB patients (n = 46) and HBV patients on antiviral treatment (n = 20). *P<.05, **P<.01, ***P<.001 by Mann-Whitney test.
(0.46 MB EPS)

Figure S3 (**A**) Representative FACS plots showing the effect of exogenous IL10 on NK cells frequencies (boxed CD56+CD3−) (**B**) NK cells from 4 eAg- CHB patients (median ALT 50, median VL 2300) were negatively purified (>96% purities) and stimulated with IL-12/IL-18 in the presence or absence of exogenous IL-10. The effect of IL-10 is shown for the CD56^bright^ subset (**P<.01 significance determined by paired t test). (**C**) Representative density plots and histograms from a healthy control and (D) a CHB patient showing NK cell IFN-γ production, gated on the CD56+CD3−TRAIL- and CD56+CD3−TRAIL+ populations, following stimulation with IL12/IL18 +/− IL-10. NK cell IFN-γ production is expressed as MFI.
(0.92 MB EPS)

Figure S4 (**A**) Production of IFN-γ by circulating and intrahepatic NK cells and NK cell subsets from 8 CHB patients with available liver samples. Paired summary bar charts expressed as mean ± SEM. (**B**) Lack of significant correlation between intrahepatic NK cell IFN-γ production and ALT. Spearman statistical test was performed (r = -0.39, p = 0.32).
(0.46 MB EPS)
